# Ultrastructural and proapoptotic-like effects of kaempferol in *Giardia duodenalis* trophozoites and bioinformatics prediction of its potential protein target

**DOI:** 10.1590/0074-02760200127

**Published:** 2020-10-21

**Authors:** Raúl Argüello-García, Fernando Calzada, Normand García-Hernández, Bibiana Chávez-Munguía, José Antonio Velázquez-Domínguez

**Affiliations:** 1 Centro de Investigación y de Estudios Avanzados-Instituto Politécnico Nacional, Departamento de Genética y Biología Molecular, Ciudad de México, México; 2 Centro Médico Nacional Siglo XXI, Unidad Médica de Alta Especialidad, Unidad de Investigación Médica en Farmacología, Ciudad de México, México; 3 Instituto Mexicano del Seguro Social, Centro Médico Nacional Siglo XXI, Unidad Médica de Alta Especialidad, Hospital de Pediatría, Unidad de Investigación Médica en Genética Humana, Ciudad de México, México; 4 Centro de Investigación y de Estudios Avanzados-Instituto Politécnico Nacional, Departamento de Infectómica y Patogénesis Molecular, Ciudad de México, México; 5 Instituto Politécnico Nacional, Escuela Nacional de Medicina y Homeopatía, Ciudad de México, México

**Keywords:** kaempferol, Giardia duodenalis, aldose reductase, reactive oxygen species, apoptosis-like

## Abstract

**BACKGROUND:**

Kaempferol (KPF) is a flavonoid with antiparasitic activity including experimental giardiasis which mechanism of action is unknown.

**OBJECTIVE:**

To analyse the cytotoxic effects of KPF on *Giardia duodenalis* trophozoites and to identify a likely parasite target of this compound.

**METHODS:**

We used inhibitory concentrations of KPF (IC_25_, IC_50_ and IC_100_) and albendazole (ABZ) as reference drug. The ultrastructure of the trophozoites was analysed by transmission electron microscopy (TEM) whilst apoptosis/necrosis, production of reactive oxygen species (ROS) and cell cycle progression were assessed by flow cytometry (FCM) and confocal laser microscopy (CLM). Ligand-protein docking analyses were carried out using KPF structure from a drug library and crystal structure of a *G. duodenalis* aldose reductase (*GdAldRed*) homolog.

**RESULTS:**

KPF provoked appearance of perinuclear and periplasmic spaces devoid of cytosolic content and multilamellar structures. KPF induced proapoptotic death associated with partial arrest in the S phase without ROS production. Bioinformatics approaches predicted that *GdAldRed* is a viable KPF target (ΔG = -7.09 kCal/mol), exhibiting 92% structural identity and a similar coupling pattern as its human homolog.

**CONCLUSIONS:**

KPF exerted a proapoptotic effect on *G. duodenalis* trophozoites involving partial interruption of DNA synthesis without oxidative stress or structure damage to chromatin and cytoskeletal structures. *GdAldRed* is a likely target underlying its antigiardial activity.

*Giardia duodenalis* (also known as *G. lamblia* or *G. intestinalis*) is an intestinal protozoan and the etiological agent of giardiasis, a diarrhoeal disease of humans and over 40 other species with the highest clinical incidences amongst parasitic diarrhoea worldwide.^1^ Giardiasis is transmitted by water or food contaminated by the infective cyst form and in humans it reaches ≈ 280 million cases per year with a prevalence of 20-30% in the developing world and 3-7% in developed countries.^2^ On the other hand, the vegetative and pathogenic trophozoites of *G. duodenalis* colonise the host’s small intestine and the clinical presentation varies from acute diarrhoea to asymptomatic and chronic outcomes. In the absence of vaccines, treatment based on a series of drugs as metronidazole, albendazole (ABZ), mebendazole and nitazoxanide may be effective in most cases although therapeutic failures might be explained by patient incompliance, suboptimal dosage as well as parasite resistance to these and other compounds.^3^ ^,^ ^4^ Therefore, it is still necessary to search new agents for therapeutic optimisation in giardiasis and natural products that include nutraceuticals are nowadays a promising source.

Within nutraceutical agents of current research, flavonoids are a large family of plant secondary metabolites characterised by a diphenylpropane structure. These are present in vegetables, seeds, fruits and beverages such as wine and beer.^5^ Their high antioxidant capacity led to the study of various protective effects against oxidative damage phenomena. It has even been reported that these active principles have various therapeutic properties to treat atherosclerosis, cancer and ischemic heart disease^6^ although from recent studies their antiprotozoal role stands out.^7^ Kaempferol (KPF) is a member of this family that has shown biological activity as antiprotozoal agent; particularly the antigiardial activity was determined using *in vitro* and *in vivo* models;^8^ ^,^ ^9^ ^,^ ^10^ however, little is known about its mechanism of action on protozoa. In this sense, preliminary *in silico* studies reported that KFP could act on the enzyme pyruvate: ferredoxin oxidoreductase.^9^ In other parasites such as *Entamoeba histolytica*, it has been suggested that this flavonoid alters functions related to structural proteins of the cytoskeleton.^11^

In order to elucidate the mechanistic basis of the cytotoxic effect of KPF on *G. duodenalis* trophozoites, a strategy based on transmission electron microscopy (TEM), flow cytometry (FCM), confocal laser microscopy (CLM) and bioinformatics analyses was carried out in the present work.

## MATERIALS AND METHODS

*Chemicals and reagents -* All reagents and solvents used were of analytical grade and were obtained from JT Baker (Mexico). ABZ (Sigma-Aldrich) was used as antiparasitic drug of reference. The test compound KPF was isolated from the leaves from *Annona cherimola* Miller (Annonaceae) collected by FC in San José, Alcaldía de Tláhuac, Mexico City, Mexico. The plant material was authenticated by MS Santiago Xolalpa of the Herbarium IMSSM of Instituto Mexicano del Seguro Social (IMSS) where the voucher specimens conserved under reference 15795. The extraction and isolation procedure were performed according to the protocol previously described by Calzada et al. ^(^ ^9^ KPF was identified by comparison [nuclear magnetic resonance (NMR), thin-layer chromatography (TLC) and high-performance liquid chromatography (HPLC)] with authentic sample disposable in our laboratory and had a high purity (> 99.99% HPLC).

*Parasites and exposure to KPF -* Trophozoites of *G. duodenalis* (WB strain) were maintained under axenic culture in Diamond’s TYI-S-33 medium modified,^12^ containing bovine bile (Sigma Chem. Co., 5 mg/mL) at 37^o^C in 4.5 mL screw-capped, flat-bottomed vials or 15 mL tubes (Falcon^TM^). Parasites were harvested by chilling flasks at the log phase of growth (usually after 72 to 96 h) for 45 min and were either counted in a haemocytometer or previously washed three times with phosphate buffered saline (PBS) and the cell concentration was adjusted as needed.

To assess the effect of KPF on trophozoites, the inhibitory concentrations (IC) at 25, 50 and 100% were used based on the reported IC_50_ value of this compound that is 30.5 µM.^8^ In all assays, 6,000 trophozoites/mL were incubated for 40 h/37^o^C in the presence of KPF previously diluted in vehicle [dimethyl sulfoxide (DMSO)] or DMSO alone (0.022% final concentration). As a positive control of cell cytoskeleton/morphology damage, ABZ was used at final concentration of 0.32 µM diluted in the same vehicle.

*TEM -* Trophozoites previously exposed to KPF at IC_100_ (61 µM) as described above were washed three times with PBS. The cell pellets were fixed with 2.5% glutaraldehyde and were subsequently fixed with 1% osmium tetroxide in cacodylate buffer. After dehydration with increasing concentrations of ethanol (from 10 to 90%) for 10 min, followed by treatment with propylene oxide: alcohol mixtures (2:1, 1:1 and 1:2) for 15 min. The preinclusion was performed using propylene oxide:resin (2:1, 1:1 and 1:2) for 15 min. Polymerisation was carried out by incubation at 60ºC for 24 h. Thin sections of 70-500 nm were cut and mounted on copper grids. Sections were stained with uranyl acetate for 15 min and lead citrate for 20 min (both from SP1 Supplies; SP1-Chem). Thin sections (approximately 30 nm thick) were observed on a JEOL 100-SX transmission electron microscope.

FCM analyses 

*Apoptosis-like/necrosis* - The presence of trophozoites exposed to KPF at IC_25_, IC_50_ and IC_100_ that displayed programmed (apoptosis-like) or spontaneous (necrosis) cell death was determined using anti-annexin V-fluorescein isothiocyanate (FITC) antibodies or propidium iodide (PI) as respective markers using a commercial kit (BioVision^TM^, USA) and following manufacturer’s instructions. In brief, PBS-washed trophozoites were incubated for 5 min at room temperature in 400 µL PBS containing 5 µL of both annexin V and PI stock solutions in the dark. The fluorescence in parasites was monitored and quantified in at least 25,000 parasites in three independent experiments using a FACSCalibur flow cytometer (Becton Dickinson^TM^) fitted with filters at 530 nm (FITC, FL1) and 585 nm (PI, FL2).

*Oxidative stress -* This method was carried out by tracing the formation of reactive oxygen species (ROS) with the fluorescent ROS-complexing agent 2´7´-dichlorodihydrofluorescein diacetate (H_2_DCFDA). Trophozoites were exposed to KPF at IC_50_ and IC_100_, washed with PBS and incubated for 30 min at 37^o^C with the fluorescent tracer at 25 µM (Image-IT^TM^ Live Green ROS Detection kit; Life Technologies, USA). Parasites were finally quantified (25,000 per sample; three independent experiments) in a FACSCalibur flow cytometer using filter at 530 nm.

*Cell cycle analysis* - To determine the proportions of trophozoites at different cell cycle stages (G0/G1, S and G2/M) after exposure to KPF at IC_25_, IC_50_ and IC_100_, the nuclei were stained with PI. In brief, parasites were washed with PBS, fixed in 70% ethanol for 30 min, washed again with PBS and incubated overnight at 4^o^C in RNAse A (0.1 mg/mL). Finally, PI was added at a final concentration of 50 µg/mL and after 30 min at room temperature samples were quantified in a FACSCalibur flow cytometer with filter at 585 nm and cell cycle stages in histogram regions were identified as reported by Reaume et al.^13^ In these assays 25,000 cells per sample (three independent experiments) were recorded.

*CLM -* A small aliquot of samples (10-15 µL) that were stained with annexin V-PI as described above were adhered to coverslips and treated with KFP (IC_25_, IC_50_ and IC_100_) for 1 h, then fixed with 4% p-formaldehyde for 1 h at 37°C, PI was subsequently added at 50 µg/mL and incubated for 15 min at room temperature. Samples were mounted with Vecta-Shield medium (Vector Laboratories, Peterborough, UK) and viewed under a Carl Zeiss LSM700 confocal microscope (Carl Zeiss, Jena, Germany) fit with laser at 480 nm.

*Bioinformatics studies -* In order to predict if KPF has the ability to interact with, and thereby inhibit, the function of giardial aldose reductase (*GdAldRed*), *in silico* approaches of protein-ligand docking were performed using the Swiss Dock web service (http://www.swissdock.ch/docking). This tool first calculates, orders in clusters and delivers a set of 256 viable positions between one rigid target *GdAldRed* and one flexible ligand (KPF). Of these predictions, the most favoured docking was selected by considering the lowest (i.e., most negative value) Gibbs free energy (∆G). In these analyses, the crystal structures of *GdAldRed* (PDB ID: 3KRB) and human aldose reductase (HsALdRed, PDB ID: 2r24) were used as *test* and reference structures respectively to compare KPF-interacting amino acids. All predictions obtained from SwissDock were visualised and edited using the UCSF chimera package v. 1.10.1. Structural comparison of the aldose reductase crystals was carried out using the TM-Align tool accessible at the I-TASSER server (https://zhanglab.ccmb.med.umich.edu/I-TASSER/).

## RESULTS

**Fig. 1:**
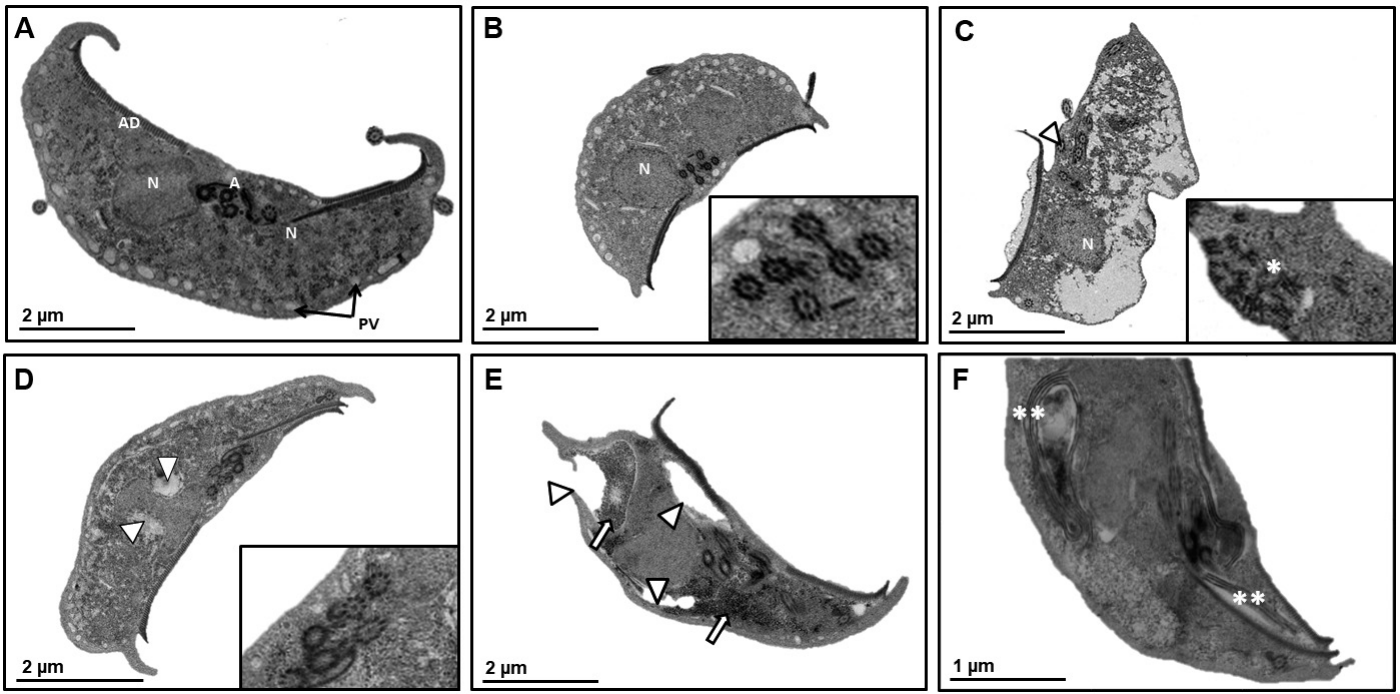
ultrastructural effects of kaempferol (KPF) in *Giardia duodenalis* trophozoites. Trophozoites of WB strain (6,000/mL) were cultured in TYI-S-33 medium (A) or exposed for 40 h/37^o^C to vehicle 0.022% dimethyl sulphoxide (DMSO) (B) as negative control, 0.32 µM albendazole (ABZ) as positive control of cytoskeletal damage (C) and KPF at IC_100_ (D-F) and processed for transmission electron microscopy (TEM). Space bar sizes are as indicated. A: flagellar axonemes; AD: adhesive ventral disk; N: nucleus; PV: peripheral vacuoles. *: fragments of AD; **: elongated multilayered structures; ∆: perinuclear and periplasmic spaces; single arrows: deposited electron-dense granules.

In a first set of experiments, TEM analyses were aimed to determine the intracellular effects of KPF on *G. duodenalis* trophozoites. In Fig. 1 panel A-B (untreated and DMSO-treated cells), trophozoite’s architecture is intact displaying the endomembranous system in cytoplasm with its typical pleomorphic peripheral vacuoles (V), rounded nuclei (N) with typical electron-density of heterochromatin and cytoskeletal elements including flagellar axonemes (A) with a 9+2 array of microtubules and ventral adhesive disk (AD) microtubules that in cross-sectional view display an ordered head-and-tail arrangement (Fig. 1B, insert). When parasites were exposed to the microtubule-acting drug ABZ,^14^ an altered cell morphology, a partial destruction of the endomembranous system (Fig. 1C), as well as the loss of organisation of AD microtubules (Fig. 1C, asterisk in insert), were clearly observed. In the case of KPF-exposed trophozoites, some different damages were noticed: irregularly shaped, large (≈ 1µm) perinuclear and periplasmic spaces devoid of electron-dense content appeared (Figs. 1D and 1E, arrowheads) that, in most cases, were adjacent to areas contoured by membranes with deposition of electron-dense granules (Fig. 1 E, arrows) and also multi lamella, elongated structures were evident (Fig. 1F, double asterisks). Unlike ABZ, KPF did not cause noticeable changes in cell morphology whilst cytoskeletal elements and nuclei shape and content seemed not altered. For instance, flagellar axonemes (A) and adhesive disk appear unaltered by this treatment (Fig. 1D, insert).

**Fig. 2:**
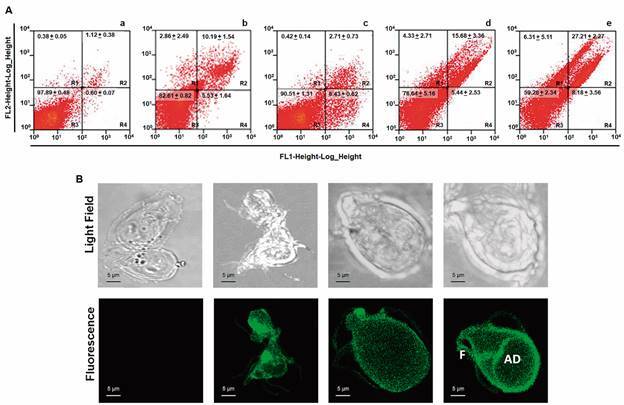
kaempferol (KPF) exerts proapoptotic-like effect on *Giardia duodenalis* trophozoites. Trophozoites of WB strain (6,000/mL) were exposed for 40 h/37^o^C to vehicle 0.022% dimethyl sulphoxide (DMSO) (a), 0.32 µM ABZ (b) or KPF at IC_25_ (c), IC_50_ (d) and IC_100_ (e) then stained with annexin V-fluorescein isothiocyanate (FITC)/propidium iodide (PI) and analysed by flow cytometry. In panel A, representative histograms are shown R1= PI fluorescence (necrosis); R2 = annexin V-PI fluorescence (late apoptosis-like); R3 = unstained cells; R4 = annexin V-FITC fluorescence (early apoptosis-like). In panel B, samples were observed under confocal laser microscope to determine the surface localisation of annexin V-associated fluorescence. Images are displayed in the following order (from left to right): 0.022% DMSO and KPF at IC_25_, IC_50_ and IC_100_. IC: inhibitor concentration.

As a multipurpose strategy, FCM studies were carried out to assess in a quantitative manner (i.e., cell-by-cell determinations) how parasites were affected by KPF at different levels. The first parameter analysed was to define the type of cytotoxicity induced by KPF as a direct (necrosis) or programmed (apoptosis-like) cell death mechanism. In these analysis, annexin V-FITC was used as a tracer to monitor the exposure of phosphatidylserine at outer cell membrane hence indicating early or late apoptosis-like (regions R4 and R2 respectively in histograms of Fig. 2A) and PI is a fluorescent dye that mainly stains nucleic acids as well as cytoplasm components due to direct damage to cell membrane (region R1). In the results shown in histograms and average counts nearly 98% cells were not stained by DMSO treatment (region R3, Fig. 2Aa). On the other hand, exposure of trophozoites to KPF at IC_25_, IC_50_ and IC_100_ induced a slight increase of parasites at early apoptosis-like (0.6 to ≈ 8.2%, region R4) and noteworthy a significant rise in late apoptotic-like cells (1.1 to 27.2%, region R2; Figs. 2Ac-2Ae). In contrast, a modest increase of cells at necrosis (≈ 0.4 to 6.3%, region R1) was observed with increasing KPF concentrations (Figs. 2Ac-e). The late apoptosis-like mechanism of KPF on *G. duodenalis* trophozoites was evidenced by the detection of phosphatidylserine at the outer cell surface of parasites by CLM, including ventral disk and flagella, whilst the vehicle DMSO alone did not cause this effect (Fig. 2B). In comparison, trophozoites exposed to the drug ABZ at its IC_100_ (0.32 µM), which causes dramatic effects in cell morphology and disrupts giardial cytoskeleton because of the direct interaction of ABZ with β-tubulin,^14^ only caused a modest increase of cells at late apoptosis-like (≈ 11%, region R2; Fig. 2Ab), in agreement with previous reports.^15^ These data are consistent with the presence of a programmed mechanism of cell death induced by KPF and are in good concurrence with the lack of disrupting effects on nucleus chromatin organisation and cytoskeletal structures as TEM studies suggested.

**Fig. 3:**
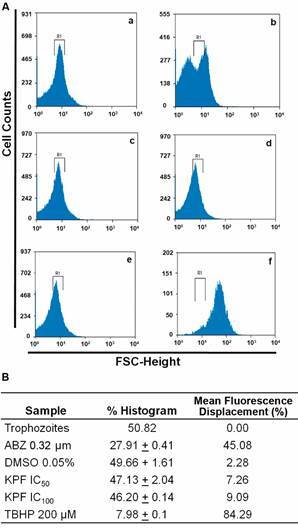
kaempferol (KPF) does not cause oxidative stress in *Giardia duodenalis* trophozoites. (A) Trophozoites of WB strain were cultured in TYI-S-33 medium (a) or exposed to, 0.32 µM albendazole (ABZ) (b), vehicle 0.022% dimethyl sulphoxide (DMSO) (c), KPF at IC_50_ (d) and IC_100_ (e) or 200 µM *tert*-butyl hydroperoxide (*TBHP*) (f), incubated with dichloro-dihydrofluorescein diacetate and reactive oxygen species (ROS) fluorescence was monitored by flow cytometry axis X: fluorescence scatter (FSC); axis Y: cell counts. R1 denotes the zone of ROS fluorescence for control samples (i.e., untreated and DMSO-treated trophozoites). (B) The percentages of cells with displaced (i.e., increased) fluorescence are indicated. Results correspond to the mean ± standard deviation of three independent experiments. IC: inhibitor concentration.

Based on the fact that KPF is a flavonoid, it was raised if its multiple hydroxyl groups could be precursors of ROS by the likely action of parasite’s enzymes able to metabolize this compound. In these assays the ROS-sensitive fluorescent tracer H_2_DCFDA was used and, as can be seen by comparing Figs. 3Aa and 3Ac (untreated and DMSO-treated trophozoites respectively) and 3Ab (ABZ treatment), this drug caused oxidative stress in agreement with other studies,^15^ as shown by a cellular fluorescence displacement of nearly 45%. Interestingly, this effect was not caused at all by KPF at IC_50_ or even at IC_100_ (Figs. 3Ad and 3Ae). As a reference, the ROS-inducing compound *tert*-butyl hydroperoxide (*TBHP*) caused a significant shift of cell fluorescence in the order of ≈ 85% (Fig. 3Af). At this point, there is robust evidence about the very distinct mechanism of cytotoxic action exerted by KPF as compared with the control drug ABZ.

**Fig. 4:**
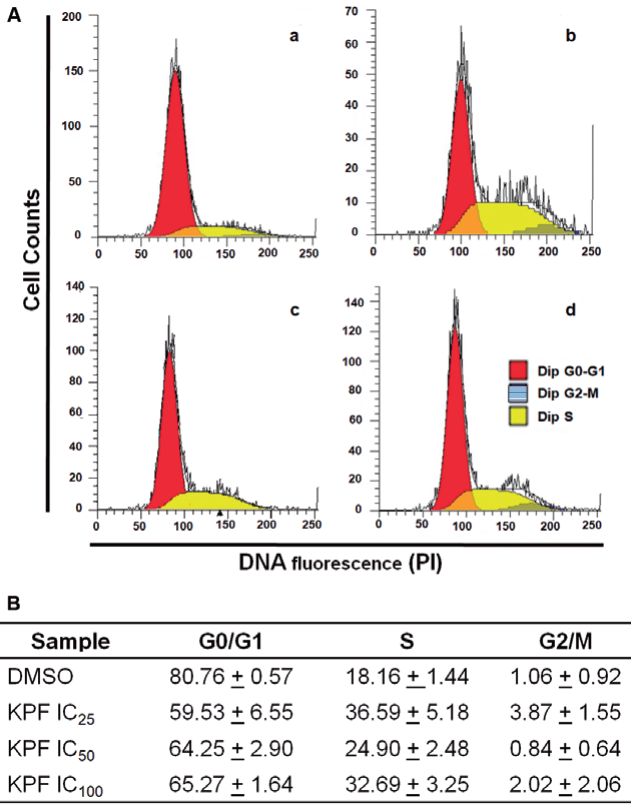
kaempferol (KPF) causes a partial arrest at S phase in *Giardia duodenalis* trophozoites. (A) Trophozoites of WB strain were exposed to vehicle 0.022% dimethyl sulphoxide (DMSO) (a) and KPF at IC_25_ (b), IC_50_ (c) and IC_100_ (d), as indicated in the corresponding histograms, then cells were treated with RNAse, nuclei were stained with propidium iodide (PI) and samples were processed by flow cytometry. Peaks corresponding to cell cycle phases are indicated at the upper right of each graph. (B) Percentages of cells at each cell cycle stage in the samples described above. Results correspond to the mean ± standard deviation of three independent experiments. IC: inhibitor concentration.

In further studies aimed to monitor dynamic changes at level of nuclear DNA, in spite that KPF apparently did not affect nuclear chromatin organisation, it was still possible that the progression of cell cycle stages (i.e., G0/G1, S, G2-M) in vegetative trophozoites that display distinct polyploidy in culture due to DNA replication (4N and 8N) could be altered by this natural flavonoid. In these analyses, trophozoite’s DNA was stained with PI and RNA was enzymatically degraded with RNAse A in cultures exposed to increasing concentrations of KPF (IC_25-100_). Histograms of such assays are shown in Fig. 4. In these, under exposure to vehicle alone (DMSO) the G0/G1 subpopulation predominated (≈80%) followed by cells at S phase (≈ 18%) with a small proportion of trophozoites at G2/M boundary (≈ 1%) (Fig. 4Aa). When KPF was increased from IC_25_ to IC_100_, a marked increase in cells at S phase was observed (24-36%) at the expense of a similar decrease of G0/G1 subpopulation (59-65%) and the G2/M subpopulation did not show significant changes (Fig. 4Ab-d). Under these conditions, there was a partial arrest of trophozoites at S phase. Together these results indicate that KPF allows G0/G1→S transit in *G. duodenalis* cell cycle and that the lack of significant changes in G2/M populations could be due to a cytostatic effect preceded by the limited previous nuclear DNA replication that in turn do not allows further cytokinesis.

In the search of a potential target of KPF in *G. duodenalis* trophozoites, the experimental data obtained allow to discard some kinds of structural biomolecules, particularly cytoskeletal proteins such as tubulins, actin, giardins among others albeit proteins associated to DNA dynamics, i.e., transcription factors, cyclins and histones could not be ruled out as likely targets of KPF. These insights also profiled cytosolic proteins, including enzymes, as target candidates. This search was simplified by virtue that in several studies, KPF and other flavonols as quercetin and polyphenols as ellagic acid, have proven inhibitory activity on the cytosolic enzyme of the polyol pathway, aldose reductase.^16^ Moreover, the interaction of KPF and its prenylated derivatives with rat lens aldose reductase along to the consequent enzyme inhibition in the low micromolar and submicromolar range was demonstrated by *in vitro* and *in silico* approaches.^17^ On the basis that in *G. duodenalis* WB strain genome two paralogs are encoded (*GdAldRed*, orfs GL50803_7260 and GL50803_9008) and that the crystal structure of the former has been experimentally solved,^18^ reliable bioinformatics studies avoiding homology modelling and stereochemical evaluations of *GdAldRed* structure were possible using the ready-to-dock structure of KPF (ZINC^12^ ID: 03869768).

**Fig. 5:**
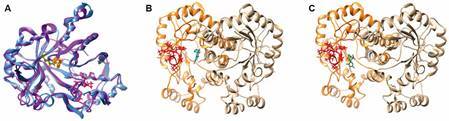
aldose reductase of *Giardia duodenalis* (*GdAldRed*) is a potential target of kaempferol (KPF) . (A) Crystal structures of *GdAldRed* (displayed in light blue) and human aldose reductase (HsALdRed) (displayed in purple) were aligned and the overall similarity is high (92%). The surface loop involved in hinge-like conformational change upon NADPH binding are displayed in ball-and-stick conformation (dark blue in *GdAldRed* and magenta in HsAldRed) whilst the residues Y48 and K77 involved in hydride transfer from NADPH to glucose are shown in green (*GdAldRed*) and in orange (HsAldRed). Protein-ligand docking was carried out with the crystal structure of *GdAldRed* dimer (monomer A displayed in orange and monomer B displayed in light brown) as receptor and the optimal docking positions are shown for KPF (B) and the specific AldRed inhibitor Fidarestat (C) that were tested as ligands and displayed in stick conformation and coloured in cyano. In *GdAldRed* structure the inhibitor-interacting domain (P303-L311) is displayed in ball-and-stick conformation and coloured in red.

In first instance, a comparison of the crystal structure of *GdAldRed* (307 amino acid long) with its human counterpart (HsAldRed, 316 amino acid long) in dimer state was carried out by structural alignment. As shown in Fig. 5A, these molecules, in spite of having 41.9% of sequence identity, share a high level of structural similarity (TM score = 0.9203) spanning 296 residues aligned with a root mean square deviation (RMSD) index of 1.63. Regarding catalytic moieties, the residues Y48 and K77 involved in hydride transfer from NADPH to glucose have identical orientation although some residues of the surface loop involved in conformational changes upon NADPH binding are differentially oriented. In general, these homologs could have similar affinity for specific ligands. In this context, the most favoured docking of KPF with *GdAldRed* was significantly favoured (∆G = -7.0941 kCal/mol) and comparable to that with HsAldRed (∆G = -7.6630 kCal/mol) involving a positioning near to the inhibitor-interacting domain (P303-L311; Fig. 5B) as occurs with the human homolog. Concurrent molecular docking analyses of *GdAldRed* with Fidarestat (ZINC^12^ ID: 5704), a potent HsAldRed inhibitor^19^ displayed a similar docking of this drug in the vicinity of the P303-L311 domain (Fig. 5C) with an optimal ∆G = -7.0697 kCal/mol, an affinity strikingly similar to that of KPF. From these bioinformatics studies, it was concluded that *GdAldRed* is a potential target of KPF.

## DISCUSSION

KPF is currently one of the most known flavonoids used as dietary supplement for healthcare because of its biphasic antioxidant properties: at low dose, it scavenges superoxide, hydroxyl and peroxynitrite species and at high doses increases the activity/expression of antioxidant enzymes such as superoxide dismutase, catalase, glutathione-peroxidase and glutathione-S-tranferase.^20^ However, there is increasing evidence about the potential pharmacological activities of KPF and some derivatives (mainly glycosides) in the control of pathologies associated to cancer, inflammation, diabetes and infectious diseases.^20^ ^,^ ^21^ In cancer, there is an increasing repertoire of molecules that have been identified to interact with or are regulated by KPF (e.g., p53, estrogen receptor, bax/bad, STAT3, caspases) that ultimately mediate antiproliferative, pro-apoptotic, pro-oxidant (i.e., ROS production) and cell cycle-arresting effects. In chronic inflammation (arthritis, allergies, atherosclerosis), KPF acts by inhibiting pro-inflammatory enzymes as are COX1/2, iNOS and modulating inflammation-related genes that include MAPK, PKC, PI3K and JAK/STAT.^22^ In diabetic complications that affect eyes, kidneys and myelin-covered tissues, there is an association of beneficial effects of KPF with the presence of the enzyme aldose reductase.^16^ Microbial pathogens as viruses are gifted with enzymes such as neuraminidase, proteases and reverse transcriptase, which activity is also inhibited by this flavonoid.^23^ In *G. duodenalis*, a deep branching eukaryote, almost all these molecules are absent or do not have an obvious homolog in its genome; nevertheless, KPF displays giardicidal activity *in vitro* and *in vivo*,^10^ hence molecular mechanisms of action and target(s) of KPF in this parasite could have distinct features.

Within the differential processes of KPF involved in its antiproliferative and proapoptotic-like effects against *G. duodenalis* trophozoites as compared to mammalian models was the absence of oxidative stress, i.e., the absence of ROS production, in contrast to the activated ROS-p38MAPK-p53 axis that precedes apoptosis in malignant melanoma and colorectal cells.^24^ Another feature was the presence of a partial arrest in S subpopulations of KPF-exposed trophozoites in this study whilst in tumoural lines as cervix HeLa and breast MCF-7/DOX cells an arrested state occurs at G2/M boundary phase.^25^ In both processes, the minimalistic genome of *G. duodenalis* explains in part why KPF does not activate ROS production nor induce a similar cell cycle arrest pattern, reinforcing the notion of a limited target repertoire.

Unlike mammalian cell models, the knowledge of mechanisms underlying the cytocidal effects of KPF or its derivatives in parasitic protozoa is still scarce. In the apicomplexan *Plasmodium falciparum* (KPF-*O*-rhamnosides and -*O*-glucosides) and the trypanosomatid *Leishmania peruviana* or *L. braziliensis* (KPF-*O*-acetyl), their activity is good (IC_50_ < 5 µM and 50-75 µM, respectively) albeit the -*O*-glucosides and -*O*-acetyl derivatives show toxicity towards macrophages or HeLa cells at around these concentrations.^26^ In the microaerophilic protozoan *E. histolytica*, KPF had an IC_50_ (36 µM) that is almost the same than that used herein in the close relative *G. duodenalis* (30.5 µM), although these values are significantly higher for glycosylated KPF derivatives and otherwise KPF itself was not toxic to mammalian cell line MT-4.^8^ ^,^ ^27^ In other studies it was observed that KPF provoked up-regulation of amoebic cytoskeletal proteins as actin, myosin II heavy chain and cortexillin II and affected some cytoskeleton-related processes of amoebic pathogenicity, i.e., diminished adhesion and increased phagocytosis and migration whilst erythrocyte binding and cytolysis were not altered.^11^ These data provide functional, albeit non pharmacodynamics, approaches to understand how KPF affects protozoan fitness, but do not preclude the possibility of similar target (s) triggering apoptotic-like pathways in *Giardia* and *Entamoeba* involving the functionality of the tubulin-rich cytoskeleton of *Giardia* or the actin-rich one of *Entamoeba*.

In this context, the use of TEM techniques aided to determine that KPF did not affected adhesive disk or flagellar tubulins nor nuclear chromatin of trophozoites in a structural landscape. Nonetheless the appearance of multilamellar structures is suggestive of autophagy as noted in studies using other compounds.^28^ Also, the use of ABZ, a drug targeting giardial β-tubulin, disassembled microtubules and served as an illustrative control of cytoskeletal effect. Congruently the pattern of cytoplasmic alterations caused by KPF and ABZ were distinct because the loss of electron-dense material left irregularly contoured “empty” areas with the former and non-contoured “granulose” areas with the latter. In addition, the proapoptotic-like effect was significantly higher with KPF (27 vs. 11%). Together, these data suggest a very distinct process leading to programmed cell death by these two compounds and open the possibility that KPF could be useful towards ABZ-resistant *Giardia*.

The final identification of *GdAldRed* as a viable target of KPF was based on: (i) previous studies about the affinity of KPF for mammalian enzyme aldose reductase;^16^ ^,^ ^17^ (ii) structure alignment analyses that confirmed the high structural similarity of *GdAldRed* (> 90%) with their human and rat homologs; and (iii) molecular docking studies that showed a similar affinity of KPF and known AldRed inhibitors as Fidarestat towards human and giardial homologs. *GdAldRed* is an enzyme involved in the NADPH-dependent conversion of glucose to sorbitol as first step in the polyol pathway and in the reduction of acetaldehyde into ethanol that otherwise is the end product of anaerobic glycolysis. Sodium valproate, a *GdAldRed* inhibitor, diminishes ethanol production and trophozoite growth.^29^ Crystal structure of *GdAldRed* was solved and found to have all domains and residues needed for catalysis.^18^ By virtue of the ubiquity of this enzyme several points are noteworthy: first, giardial and *E. histolytica* homologs share significant structural identity (88%), the amoebic homolog is also a potential target of KPF and Fidarestat (∆G = -7.04 and -7.00 kCal/mol respectively) and both parasites exhibit a similar IC_50_ towards KPF as mentioned before. Therefore, it is conceivable that AldRed is a viable common target of KPF in these organisms. Interestingly, the inhibition of mammalian AldRed that may either induce actin disassembly or microtubule formation/stabilisation^30^ is a fact consistent with that observed in *Giardia* (this work) and *Entamoeba.* ^17^ Secondly, KPF could have *in vivo* selectivity for the parasitic AldRed because *Giardia*-infected mice were successfully cured with KPF without evident secondary effects.^16^ Thirdly, *GdAldRed* is not a known target of the most commonly used drugs in giardiasis namely ABZ, metronidazole, furazolidone, nitazoxanide, paromomycin, quinacrine or reprofiled agents as auranofin^6^ thus KPF and *GdAldRed* could be considered even as potential agent and molecular target against drug-resistant *Giardia*.

In conclusion, this work suggests that KPF has an antiproliferative, proapoptotic-like mode of action against *G. duodenalis* that differs from that observed in mammalian cells, viral pathogens or even other protozoa although in this regard it could be similar to closely related pathogens as *E. histolytica*. The evaluation of *GdAldRed* as a molecular target of KPF certainly deserves further studies and opens new perspectives about the utility of this molecule as a new drug target in this and other parasites. Importantly, the spectrum of health benefits of KPF as an AldRed inhibitor could be considered as double-edged: on one hand, as a promising agent for treatment of diabetic complications; on the other, as an effective anti-infectious agent. From the perspective of proactive medicine, these notions support repurposing some dietary supplements into pharmaceutically functional agents.

## References

[1] Einarsson E, Ma'ayeh E.Svärd SG (2016). An up-date on Giardia and giardiasis, Curr Opin. Microbiol.

[2] Ryan U, Hijjawi N, Feng Y, Xiao L (2019). Giardia an under-reported foodborne parasite. Int J Parasitol.

[3] Argüello-García R, Cruz-Soto M, Romero-Montoya L, Ortega-Pierres G. (2004). Variability and variation in drug susceptibility among Giardia duodenalis isolates and clones exposed to 5-nitroimidazoles and benzimidazoles in vitro.. J Antimicrob Chemother.

[4] Leitsch D (2015). Drug resistance in the microaerophilic parasite Giardia lamblia. Curr Trop Med Rep.

[5] Hertog MGL, Hollman PCH, van de Putte B (1993). Content of potentially anticarcinogenic flavonoids of tea, infusions, wines, and fruit juices. J Agric Food Chem.

[6] Jang M, Cai L, Udeani GO, Slowing KV, Thomas CF, Beecher CWW (1997). Cancer chemopreventive activity of resveratrol, a natural product derived from grapes. Science.

[7] Soto J, Gómez C, Calzada F, Ramírez ME (2010). Ultrastructural changes on Entamoeba histolytica HM1-IMSS caused by the flavan-3-ol, (-)-epicatechin. Plant Med.

[8] Calzada F, Alanis DA, Meckes M, Tapia-Contreras A, Cedillo-Ribera R (1998). In vitro susceptibility of Entamoheba histolytica and Giardia lamblia to some medical plants used by the people of southern Mexico. Phyt Res.

[9] Calzada F, Correa-Basurto J, Barbosa E, Mendez-Luna D, Yepez-Mulia L (2017). Antiprotozoal constituents from Annona cherimola Miller, a plant used in Mexican traditional medicine for the treatment of diarrhea and dysentery. Phar Mag.

[10] Barbosa E, Calzada F, Campos R (2007). In vivo antigiardial activity of three flavonoids isolated of some medicinal plants used in Mexican traditional medicine for the treatment of diarrhea. J Ethnopharmacol.

[11] Bolaños V, Diaz-Martinez A, Soto J, Marchat LA, Sanchez-Monroy V, Ramirez-Moreno E (2015). Kaempferol inhibits Entamoeba histolytica growth by altering cytoskeletal functions. Mol Biochem Parasitol.

[12] Keister DB (1983). Axenic culture of Giardia lamblia in TYI-S-33 medium supplemented with bile. Trans of The Royal Society of Tropical Medicine and Hygiene.

[13] Reaume C, Moore B, Hernández P, Ruzzini A, Chlebus M (2013). Ion of drugs and stationary growth on the cell cycle of Giardia intestinalis Mol. Biochem. Parasitol.

[14] Chávez B, Cedillo-Rivera R (1992). Giardia lamblia ultrastructural study of the in vitro effect of benzimidazoles. J Protozool.

[15] Martínez-Espinosa R, Argüello-García R, Saavedra E, Ortega-Pierres G (2015). Albendazole induces oxidative stress and DNA damage in the parasitic protozoan Giardia duodenalis. Front Microbiol.

[16] Veeresham C, Rao AR, Asres K (2014). Aldose reductase inhibitors of plant origin. Phytother Res.

[17] Jung HA, Moon HE, Oh SH, Kim BW, Sohn HS, Choi JS (2012). Kinetics and molecular docking studies of kaempferol and its prenylated derivatives as aldose reductase inhibitors. Chem Biol Interact.

[18] Ferrell M, Abendroth J, Zhang Y, Sankaran B, Edwards TE, Staker BL (2011). Structure of aldose reductase from Giardia lamblia. Acta Crystallogr Sect F Struct Biol Cryst Commun.

[19] Oka M, Matsumoto Y, Sugiyama S, Tsuruta N, Matsushima M (2000). A potent aldose reductase inhibitor, (2S,4S)-6-fluoro-2', 5'-dioxospiro[chroman-4,4'-imidazolidine]-2-carboxamide (Fidarestat) its absolute configuration and interactions with the aldose reductase by X-ray crystallography. J Med Chem.

[20] Rajendran P, Rengarajan T, Nandakumar N, Palaniswami R, Nishigaki Y (2014). Kaempferol, a potential cytostatic and cure for inflammatory disorders. Eur J Med Chem.

[21] Kim SH, Choi KC (2013). Anti-cancer effect and underlying mechanism(s) of kaempferol, a phytoestrogen, on the regulation of apoptosis-like in diverse cancer cell models. Toxicol Res.

[22] Devi KP, Malar DS, Nabavi SF, Sureda A, Xiao J, Nabavi SM (2015). Kaempferol and inflammation from chemistry to medicine. Pharmacol Res.

[23] Calderón-Montaño JM, Burgos-Morón E, Pérez-Guerrero C, López-Lázaro M (2011). A review on the dietary flavonoid kaempferol. Mini Rev Med Chem.

[24] Choi JB, Kim JH, Lee H, Pak JN, Shim BS, Kim HS (2018). Reactive oxygen species and p53 mediated activation of p38 and caspases is critically involved in kaempferol induced apoptosis-like in colorectal cancer cells. J Agric Food Chem.

[25] Zhang Q, Zhao X-H, Wang Z-J (2009). Cytotoxicity of flavones and flavonols to a human esophageal squamous cell carcinoma cell line (KYSE-510) by induction of G2/M arrest and apoptosis-like. Toxicol in Vitro.

[26] Cai S, Risinger AL, Nai-r S, Peng J, Anderson TJ, Du L (2016). Identification of compounds with efficacy against malaria parasites from common North American plants. J Nat Prod.

[27] Cimanga RK, Kambu K, Tona L, Hermans N, Apers S, Totte J et al (2006). Cytotoxicity and in vitro susceptibility of Entamoeba histolytica to Morinda morindoides leaf extracts and its isolated constituents. J Ethnopharmacol.

[28] Matadamas-Martínez F, Castillo R, Hernández-Campos A, Méndez-Cuesta C, de Souza W, Gadelha AP (2016). Proteomic and ultrastructural analysis of the effect of a new nitazoxanide-N-methyl-1H-benzimidazole hybrid against Giardia intestinalis. Res Vet Sci.

[29] Schofield PJ, Edwards MR (1991). Glucose metabolism in Giardia intestinalis. Mol Biochem Parasitol.

[30] Rivelli JF, Amaiden MR, Monesterolo NE, Previtali G, Santander VS, Fernández A (2012). High glucose levels induce inhibition of Na, K-ATPase via stimulation of aldose reductase, formation of microtubules and formation of an acetylated tubulin/Na,K-ATPase complex. Int J Biochem Cell Biol.

